# Dynamics of Trimming the Content of Face Representations for Categorization in the Brain

**DOI:** 10.1371/journal.pcbi.1000561

**Published:** 2009-11-13

**Authors:** Nicola J. van Rijsbergen, Philippe G. Schyns

**Affiliations:** 1 Department of Psychology, University of Glasgow, Glasgow, United Kingdom; 2 Centre for Cognitive Neuroimaging, University of Glasgow, Glasgow, United Kingdom; University College London, United Kingdom

## Abstract

To understand visual cognition, it is imperative to determine when, how and with what information the human brain categorizes the visual input. Visual categorization consistently involves at least an early and a late stage: the occipito-temporal N170 event related potential related to stimulus encoding and the parietal P300 involved in perceptual decisions. Here we sought to understand how the brain globally transforms its representations of face categories from their early encoding to the later decision stage over the 400 ms time window encompassing the N170 and P300 brain events. We applied classification image techniques to the behavioral and electroencephalographic data of three observers who categorized seven facial expressions of emotion and report two main findings: (1) over the 400 ms time course, processing of facial features initially spreads bilaterally across the left and right occipito-temporal regions to dynamically converge onto the centro-parietal region; (2) concurrently, information processing gradually shifts from encoding common face features across all spatial scales (e.g., the eyes) to representing only the finer scales of the diagnostic features that are richer in useful information for behavior (e.g., the wide opened eyes in ‘fear’; the detailed mouth in ‘happy’). Our findings suggest that the brain refines its diagnostic representations of visual categories over the first 400 ms of processing by trimming a thorough encoding of features over the N170, to leave only the detailed information important for perceptual decisions over the P300.

## Author Summary

How the brain uses visual information to construct representations of categories is a central question of cognitive neuroscience. With our methods we visualize how the brain transforms its representations of facial expressions. Using electroencephalographic data, we analyze how representations change over the first 450 ms of processing both in feature content (e.g., which aspects of the face, such as the eyes or the mouth are represented across time) and level of detail. We show that facial expressions are initially encoded with most of their features (i.e., mouth and eyes) across all levels of details in the occipito-temporal regions. In a later phase, we show that a gradual reorganization of representations occurs, whereby only task relevant face features are kept (e.g., the mouth in “happy”) at only the finest level of details. We describe this elimination of irrelevant and redundant information as ‘trimming’. We suggest that this may be an example of the brain optimizing categorical representations.

## Introduction

How visual representations evolve over time in the brain remains a challenge for cognitive neuroscience. We know from psychophysics and neuroscience that the early stages of vision analyze input across a bank of spatial filters [1]–[3]. These are thought to produce complete representations of visual events at different levels of detail for higher-level categorization processes. Although this idea has become commonplace, it still remains unknown how representations transform from a thorough analysis of the retinal input into a construct that separates the irrelevant details of the environment from the features that are critical for the categorization task [4].

Early research focused on the respective roles of the coarse vs. fine scale information (technically, the Low vs. High Spatial Frequencies, LSF vs. HSF) for visual categorization. It was thought that coarse scales (i.e. LSF) had general priority [5], through activating intrinsically faster pathways (i.e. magno-cellular [6],[7]), through representing more detectable information (i.e. with higher LSF contrast energy), or through engaging automatic mechanisms adapted by evolution to detect important events (e.g. threatening stimuli [8],[9]), or an interaction of these main factors [10],[11]. Recent findings suggested that the visual system could instead be more opportunistic, initially biased by task, or context, to give immediate priority to the information needed to categorize the input (i.e. diagnostic information), at whatever level of detail that information is represented [12]–[14]. This change of emphasis from a fixed use of information from early spatial filters to a task-dependent, flexible account of encoding raises a number of critical questions: How does information from all spatial filters become analyzed and combined into a categorization-supporting construct? Does the construct remain stable over time, and a faithful representation of the initial inputs? Or does it evolve over time to optimize the categorization task at hand?

We framed these questions about the dynamics of visual representations in the context of ecologically important categorizations for the human species: the six Ekman facial expressions of emotion plus “neutral.” Facial expressions and spatial scales are known to modulate the amplitude and phase of the face-sensitive cortical response N170 [15]–[23]. However, this event does not mark the end of the categorization process. Up to 200 ms of further processing leads to a positive component with an onset around 300 ms (conventionally referred to as a P300), another cortical event modulated by categorization task [23]–[25]. So, we can think of the entire N170 deflection and the P300 response as bracketing a global process that transforms a face stimulus encoded across the Spatial Frequencies (SF) impinging on the retina into a categorized face represented in a format yet to be discovered. Capturing when and how this transformation takes place in the brain is paramount to understanding when and how the brain transforms the visual input into category-specific representations.

In the experiment, observers performed a 7–choice expression categorization task of FACS-coded faces [26] randomly presented one at a time on the CRT monitor: “neutral,” “happy,” “surprise,” “fear,” “disgust,” “anger,” or “sad”. On each trial, we randomly sampled visual information in five non-overlapping SF bands of one octave each using the “Bubbles” technique. In each trial, the random sampling revealed sparse information from a given facial expression [27] (see Methods, Stimuli). Concurrently, we measured the response of the brain to the sparse information by measuring the electroencephalographic (EEG) activity on 58 scalp electrodes, with a 3.9 ms time resolution [17] (see Methods, Procedure and EEG Recording).

In the ensuing analysis, for each observer we regressed the sampled information to their behavioral and EEG data, across five SF bands used in sampling (see Methods, Computation). This analysis isolated the information subset correlated with behavioral and EEG responses. For each band, we quantified information in terms of cycles per face (see Methods, Information in SF bands).

For behavior, the outcome is one classification image per observer and expression; for the EEG, the outcomes are 115 3.9 ms classification images, for each of the 58 electrodes. From the conjunction of EEG and behavioral classification images information one can track how the facial features important for accurate categorization of facial expressions are initially encoded and then dynamically transformed in the brain for categorical decision.

For the first time, we broadly characterized the overall dynamic transformations of the first 400 ms of feature processing along the two main dimensions of information sampled with Bubbles. First, in terms of the feature content (e.g. the eyes, the nose or the mouth) that appears at different time points of the EEG signal. Second, in terms of the specific combinations of SF bands that represents this feature content at each time point.

## Results/Discussion

We performed analyses of feature content and SF composition on all 58 electrodes and time points. To reduce dimensionality, we selected a subset of 19 electrodes that covered the entire scalp topography (see Methods, Computation: Sensor-Based EEG Classification Images). Our detailed analysis concerns the 3 electrodes that best reflected the overall dynamics of change over the first 400 ms of processing: the Left PO7 and Right P8 Occipito-Temporal electrodes (OTL and OTR, where the N170 reached maximum amplitude) and parietal electrode POz (where the P300 peaks). These three sensors consistently had the highest EEG and information peaks throughout the analyzed time course. In addition, we generalized the results to the entire scalp and all expressions performing a global analysis on the subset of 19 electrodes (including OTR, OTL and POz).

Our results illustrate that a first occipito-temporal process coinciding with the N170 time course encodes facial expressions with most of their feature content, using combinations of all SF bands. Information processing then moves to parietal regions where this thorough encoding is trimmed of the redundant Low SF bands, leaving a detailed HSF representation of diagnostic features over the P300.

### Representational Change: Feature Content

**Figure 1:**
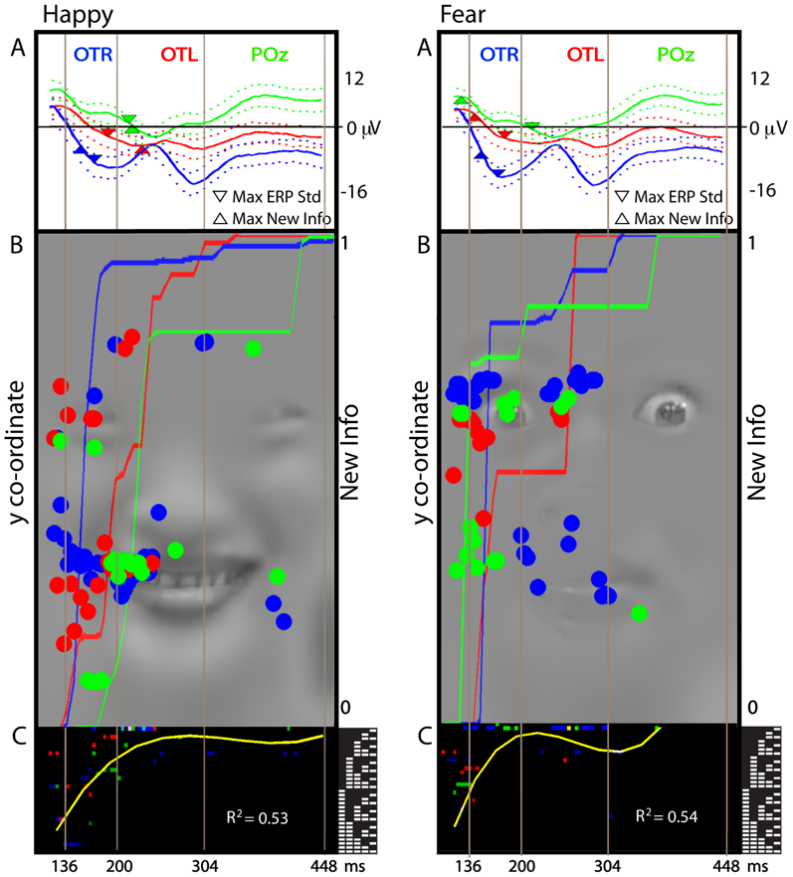
Trimming representations of diagnostic facial features (observer LP, ‘happy’ and ‘fear’). Panel A: Average EEG. Colored curves represent the average EEG over PO7 (OTL, red), P8 (OTR, blue) and POz (green), while dashed curves represent the variance of the EEG. The upward triangle represents the point of maximum of EEG variance. On OTR and OTL this point is close to the upside-down triangle, (the maximum of the derivative of information accumulation shown on Panel B) where most novel, behaviorally relevant information is added to the classification images. Panel B: Behavior and Brain Feature Content. The gray-level background faces represent the facial features required for categorization behavior. Color-coded dots represent OTL and OTR sensors in red and blue; POz in green. Their Y coordinates, in correspondence with the behavioral features, reveal the location of the maximum of information of the EEG classification image at each time point. The color-coded curves plot the time course of accumulation of new information relevant for behaviour on each electrode. Most new information arrives during the early N170, indicating that most processing after the N170 is re-organization of inputs already taken up. Panel C: Spatial Frequency Composition. For each dot of panel B, a corresponding colored dot represents the binary-coded combination of SF bands of this particular feature at this time point. The colored dots trace a systematic upward trajectory over time in the binary codes, summarized, in yellow, with a cubic fit to those points containing diagnostic information. The trajectories illustrate that the same diagnostic features evolve from involving most SF bands (between 136 and 200 ms) to involving only the highest SF bands (between 200 and 448 ms).

In Figure 1, we illustrate the development of sensitivity to features over time for the expressions Happy and Fear, starting with the signal. Panel A of Figure 1 represents the time course of the average and variance of the EEG response to “happy” and “fear” (color-coded in blue for OTR, in red for OTL and in green for POz). On Panel B we show the location of the face features the EEG is sensitive to, over time. Using the same color-code, for the same electrodes and time points Panel B represents with a dot the maximum of featural information in the corresponding EEG classification images (see Methods, Computation: Sensor-Based EEG Classification Images, Information Maximum). The Y-coordinate of the maximum in pixel space indicates feature content, and the X-coordinate corresponds to the time after stimulus onset. The background facial expressions are the behavioral classification images representing the facial features that Observer LP used to correctly classify this expression—i.e. the outside corners of the eyes and the mouth revealing the teeth for “happy” vs. the wide-opened eyes revealing the white for “fear.” Panel B therefore illustrates that the feature content represented in the EEG signal (i.e. the colored dots) changes over time to represent the entire feature content (e.g. the eyes and the mouth in “happy”) necessary for correct behavioral categorization response (as shown the background facial expression). The overlap between the colored dots and the gray-level background expression makes this point.

To illustrate, consider the first time window elapsing between 136 and 200 ms (isolated by the first two vertical bars). In “happy,” blue and red dots initially located on the eye region and then the blue and red dots located on the mouth region reveal that OTR and OTL are sequentially sensitive to these features over the N170 time course [17]. In “fear,” the same dots are mostly located on the eyes. Within this initial time window, the POz green dots fall on both the eyes and the mouth, for both expressions. The second time window changes this pattern. Starting at about 200 ms, feature content shifts more clearly to diagnostic information.

It is interesting to note the repetition of the same feature content over time—as represented by the similar Y-coordinate locations of colored dots in panel B. This suggests that novel diagnostic features are only extracted over a certain time period to be further processed afterwards. To underline this point, we computed the time course of the addition of novel, behaviorally relevant information (see Methods, Computation: Comparison of EEG and Behavioral Classification Images). The color-coded cumulative curves in Panel B of Figure 1 represent this integration process. The curves illustrate that most novel information is added early, in the time window of the N170, before the N170 peak (as marked as a triangle on Panel A, near the point of maximum variance in the ERP, marked with an upside-down triangle). Between 200 and 448 ms the cumulative curves flatten implying that little novel information enters the EEG classification images.

In sum, the first 400 ms suggest a global transition between two main phases of processing of feature content. In a phase elapsing between the first 136 to 200 ms, OTR and OTL electrodes are initially sensitive to the eyes and then move down on the face to the diagnostic, expression-specific features when the N170 peaks [17]. This period corresponds to the appearance of most novel features in the EEG classification images, suggesting their extraction from the input. In the following time window elapsing between 200 and 448 ms, sensitivity to the same diagnostic features remains, but processing moves from the occipito-temporal to the parietal regions (green dots) over the time course of the P300 associated with perceptual decision [24],[28]. We now examine how the representation of the features, in terms of their spatial frequency composition, transforms over time.

### Representational Change: Spatial Frequency Composition

We have shown that the dynamics of feature content evolves from occipito-temporal to parietal regions. As explained, we know that early vision decomposes the input stimulus, including diagnostic features, into a full representation of the SFs impinging on the retina. But as the behavioral classification images illustrate (see Figure 1, Panel B), categorization of “happy” only requires the wrinkled corners of the eyes and the mouth revealing the teeth. These are fine resolution features. Their information will be fully represented in HSF, but with little contrast, due to the 1/f^2^ decrease in contrast energy typical of face stimuli [29],[30]. Given that the early stages of visual processing are more sensitive to contrast which facilitates their detection and extraction, the later processing stages could be comparatively more sensitive to the rich information content of HSF important for perceptual decision and less so the redundant lower SF representations of features. We should therefore expect a “SF trimming” of the redundant SF components of diagnostic representations between the end of the N170 and later perceptual decision. This could reflect a reorganization and refinement of facial features following their occipito-temporal encodings.

To examine this, we plotted in Panel C, for each colored point of panel B, the corresponding combination of SF bands representing this particular feature with a binary code (where 11111 means “all SF bands” and 0001 means “only the highest SF band”; see the binary coding to the right of Panel C, and Figure S1 for examples; see Methods. Computation: Spatial Frequency Coding). For both expressions and electrodes, the colored binary codes trace a systematic upward trajectory over time. This trajectory, fitted with a cubic polynomial represented in yellow, reflects a change in representations from involving combinations of most SF bands in the first N170 time window, to representations involving only the highest SF bands in the time window leading to the P300. This second phase of processing appears to reflect a refinement of facial features already extracted from the inputs (cf. the cumulative curve of novel information peaking at the end of the N170). Figure S5 illustrates a complementary analysis of the trimming process by examining the sensitivity of the EEG time course to information in each SF band, revealing that LSF band information is gradually eliminated.

Taken together, the analysis of both feature and SF dimensions, suggest that the same diagnostic features represented in broadband spatial frequency combinations during the N170 are represented in higher spatial frequencies during the P300.

### Generalization to Seven Expressions

**Figure 2:**
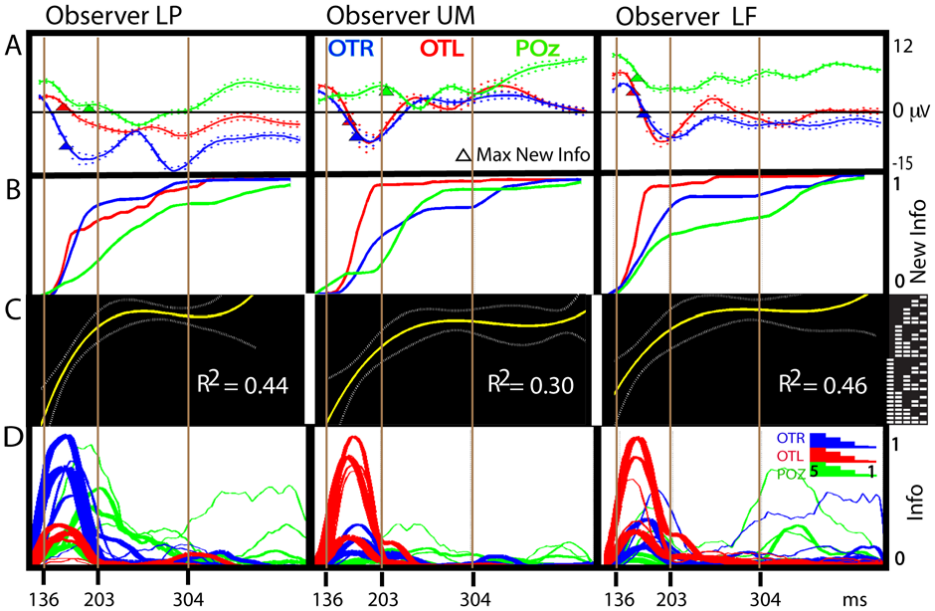
Trimming representations of diagnostic facial features (all observers, averages across 7 expressions). Panel A: Average EEG. The colored curves represent the average EEG of the seven expressions over Occipito-Temporal sensor PO7 (OTL, in red), P8 (OTR, in blue) and Parietal POz (POz, in green). Surrounding dashed curves represent the variance of the EEG at these time points. We also represent the point where most novel information is added, on average. Panel B: Average Cumulative New Information. Panel B shows the cumulative sum of new, behaviorally relevant information on each electrode, summed for each expression, and then averaged. OTR and OTL take up almost all information before the end of the N170. Panel C: Spatial Frequency Composition. For each observer, we averaged the cubic polynomial fit derived for each of the seven expressions shown during the experiment (see Panel B of Figure 1 and Figure S3 and S4 for specific examples). We represented the average trajectories in time over the binary coding space (with variance represented as dashed lines). The trajectories illustrate the nonlinear trimming of spatial frequency content of feature representations in the brain from full signal (high-numbered binary codes) before 200 ms (corresponding to processing in the occipito-temporal regions) to only higher spatial frequency content (low-numbered binary codes) after 200 ms (corresponding to parietal processing). Panel D: Facial Information Content per Spatial Frequency Band. For each observer, we also averaged across the seven expressions the respective contribution of each spatial frequency band (thickest line represents lowest Spatial Frequency band). Line height illustrates the quantity of information (expressed in cycles per face) present in the classification image at this time point. This panel illustrates that all Spatial Frequency bands initially contribute information on the OTL and OTR (in red and blue) classification images. In contrast, only higher SFs contribute information to the late classification images on POz (in green).

In Figure 2, we generalize these findings by reporting for each observer the results computed independently for each expression (n = 7) and then averaged. Panel A presents the average EEG and variance for each electrode. Panel B the average cumulative function of new features. Panel C the average cubic fits and Panel D the averaged total facial information content per SF band (see Methods, computation of SF information). This generalization across all seven expressions illustrates for each observer the occipito-temporal integration of novel information across all SF bands over the N170 time course, followed by a parietal transformation of the information over the P300 time course. This transformation trims the LSF bands, emphasizing HSF information. Panels B to D jointly illustrate that whereas little new information is added after the N170 time period, the remaining information is thinned.

### Generalization to 19 Electrodes Covering the Whole Scalp

So far, our results arose from the three electrodes characteristic of the overall dynamics of the first 400 ms of information processing. To establish that the reported trimming process characterizes the overall evolution of brain representations, we generalize the analysis and results from three specific electrodes to 19 electrodes covering the whole scalp topography. To simplify the dimensionality of feature content, spatial frequency composition and time course, we followed the following steps: For feature content, we defined three main regions of interest that are recurrently diagnostic of the seven expressions: the left eye, the right eye, and the mouth. For spatial frequency composition, we classified the combinations of SF bands representing the features into two categories: High (any combination of the first three SF bands; binary code 1 to 7) and Broad (any other combination, binary code 8 to 31). For the time course, we divided the first 450 ms into four distinct time intervals.

For each observer, collapsing across all seven expressions, we computed, for each time interval, the number of times the EEG classification images of the 19 electrodes represented the diagnostic features of interest and when they did so, we classified their spatial frequency content as either High or Broad. For each feature, we represented the frequency of these encodings over each time period as the radius of a circle (empty for High; filled for Broad). The expanding empty circles (High) together with the contraction of filled circles (Broad) illustrate the trimming process. Trimming, the shift from Broad spectrum to HSF representation of diagnostic features therefore characterizes the overall brain dynamics, not just a few well-chosen electrodes.

To conclude, we report two main findings on the transformation of feature representations in terms of content and SF composition that were robust across all three observers and seven expressions. First, the representation of diagnostic features evolves from an initial stage of encoding in the occipito-temporal electrodes to a later stage of perceptual reorganization on the parietal electrode. These two stages appear to correspond in time to the N170 and the P300 ERPs. Second, while this occipito-temporal to parietal shift of information processing happens, the same diagnostic representations change in SF composition and quantity of information from broadband and thorough in the occipito-temporal regions to sparse and rich in HSF information in the parietal region, supporting the idea of a refinement and reorganization of facial features.

### Conclusion

We argued that the understanding of visual cognition implies an understanding of when, how and with what information the brain categorizes its visual inputs. In the context of a biologically relevant task–the categorization of seven facial expressions of emotion–we have shown how the brain transforms its visual inputs (i.e. spatial frequencies impinging on the retina) into a categorization-supporting construct. In three observers, we found that information sensitivity shifts from the occipito-temporal to the parietal regions between 138 and 448 ms following stimulus onset. While this happens, visual representations transform from full signal encodings of diagnostic features during the N170 time course, when most new features are acquired, to a representation preserving only HSF details of the same features following the N170 peak. This characterization was true for all observers and input categories.

The transformation from full signal to HSF during the N170, followed by a progressive trimming of HSF information through the P300, combined with the observation that little new information is taken up after the N170 peaks, consistent with very rapid encoding [31]–[33], suggests a division of function between the two phases. From 140 to 200ms, the N170 is primarily concerned with the take up of information in the stimulus. This early phase is concerned with generic face information, but close to the peak becomes biased towards HSF, and expression specific (i.e. diagnostic) information. It is tempting to associate this information dynamics with spatial attention and stimulus encoding, both in terms of the two-dimensional locations of features on the face, but also in terms of the SF composition of these features in a third dimension of spatial scales. Note that the exact contribution of SFs may depend on the scale of the diagnostic information itself. Thus, as a methodological note, examining generic sensitivity to SFs over this time interval might not be reliably informative about the N170. In the later time interval, from 200 to 448ms, the information transfer between occipital temporal areas and parietal areas appears to involve the reorganization of the representation, and favors information at finer spatial scales. It is tempting to interpret these dynamics as an example of the brain optimizing its internal representations for decision, perhaps involving memory storage [34]–[36]. Variability in the P300 component has been related to active processes such as evidence evaluation [24], working memory load [37] as well as categorization [38] However, we cannot rule out the possibility that trimming may be a passive process, unrelated to selection [34].

### Broad-to-Fine Processing

Our results apparently contradict the typical coarse-to-fine processing reported in psychophysics [5] and visual cognition [9],[10] (and see [39] for discussions of alternative dynamics). In coarse-to-fine processing, an initial skeleton of LSF information is subsequently fleshed out with HSF featural details. Our data suggest instead a Broad-to-Fine process, in which High and Low SF information are processed equally up to the peak of the N170 (which coincides with the peak of integration of novel features, see Figure 2 and Figure S5, and S6), followed by selective processing of HSF information. In particular, Figure S6 shows that there is no bias towards LSF information over the N170 [40].

The timing of the observed broad-to-fine processes appears to be more consistent with a late (as opposed to early) selection model of attention. However, the early integration of specific SF features over the N170, from the eyes down on the face, rules out their late selection, suggesting their early encoding on the N170. This suggests a more complex model of neural information encoding, in which the categorization task of the observer controls early N170 encodings of diagnostic features across the broad SF spectrum. These broad spectrum encodings are then trimmed to preserve their HSF components over the time course leading to the P300. Full-to-fine processing is therefore compatible with an early, diagnostic selection of featural information, but a late selection of SF information. But if this is dependent on the categorization task of the observer, early selection of SF information may be possible, if the categorization task requires only this information. To conclude, the evidence for rapid selection of an initial diagnostic feature set (over the N170 time course) [31]–[33] is persuasive.

Although we characterize the changes through the N170 topography to the P300 as a process of information transfer, one of the limitations of our methods is that we rely on a statistical relationship with the input space, rather than on a direct relationship between the early encoding phase and late phase. A method such as Granger Causality, applied directly to the signal might reveal the strength of the direct relationship. Our prediction, based on the analyses here, would be that strong interactions between sources driving the initial encoding, and those underlying the later phase are likely to be associated with successful transfer of HSF diagnostic features. This transfer may be part of the process of selection of task relevant information, or it may precede the processes of selection.

From our data set, as with many EEG studies, we cannot reliably make inferences about the precise sources underlying the reported SF dynamics and trimming of representations. However, evidence from brain imaging (e.g. [14]) suggests that the Fusiform Face Area, one of the sources of the N170 [41], and possibly the N250 [42] is also a zone of convergence between HSF and LSF information, and this may also be the first stage in the ‘trimming’ process. We can however, say little, as yet, about the possible sources of the later stage. However, we note that the reorganization may also sometimes involve the reactivation of information occipital temporal areas during the later phase, suggesting that contemporaneous representation of closely related information can be spread over multiple locations and presumably multiple sources [41]–[43]. Localization of sources and interpretation of their interactions will form much of our future work.

In summary, our data show the process of transformation from a thorough visual representation that accurately reflects the structure of the retinal input, to a sparser representation that reflects the informational structure of the category.

## Methods

### Observers

Three University of Glasgow observers (LP, LF and UM) were paid to take part in the experiment.

### Ethics Statement

All three observers had normal vision and gave informed consent prior to involvement. Glasgow University Faculty of Information and Mathematical Sciences Ethics Committee provided ethical approval.

### Stimuli

Original stimuli were gray-scale images of five females and five males taken under standardized illumination, each displaying seven facial expressions of emotion. The resulting 70 stimuli (normalized for the location of the nose and mouth) complied with the Facial Action Coding System (FACS [26]), and form part of the California Facial Expressions (CAFE) database. As facial information is represented at multiple spatial scales, on each trial we exposed the visual system to a random subset of SF information contained within the original face image. To this end, we first decomposed the original image into five non-overlapping SF bands of one octave each (120−60, 60−30, 30−15, 15−7.5 and 7.5−3.8 cycles/face). To each SF band, we then applied a mask punctured with Gaussian apertures. The size of the apertures was adjusted for each SF band, so as to reveal 6 cycles per face (standard deviations of the bubbles were 0.36, 0.7, 1.4, 2.9, 5.1 cycles/degree of visual angle from the fine to the coarse SF band). Apertures were positioned in random locations trial by trial, approximating a uniform sampling of all face regions across trials. Calibration of the sampling density (i.e. the number of bubbles sampling the face on each trial) was performed online on a trial-by-trial basis, to maintain observer's performance at 75% correct categorization independently for each expression. The stimulus presented on each trial comprised the randomly sampled information from each SF band summed together (see Panel A of Figure S1. for an illustration of the stimulus generation process).

### Procedure

Prior to testing, observers learned to categorize the 70 original images into the 7 expression categories. Upon achieving a 95% correct classification criterion of the original images, observers performed a total of 15 sessions of 1400 trials (for a total of 21,000 trials) of the facial expressions categorization task (i.e. 3000 trials per expression, happy, sad, fearful, angry, surprised, disgusted and neutral faces, randomly distributed across sessions). Short breaks were permitted every 100 trials of the experiment.

In each trial a 500 ms fixation cross (spanning 0.4° of visual angle) was immediately followed by the sampled face information, as described above (see Figure S1). Stimuli were presented on a light gray background in the centre of a monitor; a chin-rest maintained a fixed viewing distance of 1 m (visual angle 5.36°×3.7° forehead to base of chin). Stimuli remained on screen until response. Observers were asked to respond as quickly and accurately as possible by pressing expression-specific response keys (7 in total) on a computer keyboard. The experiment was programmed with the Psychophysics Toolbox for Matlab [44],[45].

### EEG Recording

We used sintered Ag/AgCl electrodes mounted in a 62-electrode cap (Easy-Cap) at scalp positions including the standard 10–20 system positions along with intermediate positions and an additional row of low occipital electrodes. Linked mastoids served as initial common reference, and electrode AFz as the ground. Vertical electro-oculogram (vEOG) was bipolarly registered above and below the dominant eye and the horizontal electro-oculogram (hEOG) between the outer canthi of both eyes. Electrode impedance was maintained below 10 kΩ throughout recording. Electrical activity was continuously sampled at 1024 Hz. Analysis epochs were generated off-line, beginning 500 ms prior to stimulus onset and lasting for 1500 ms in total. We rejected EEG and EOG artifacts using a [−30 µV; +30 µV] deviation threshold over 200 ms intervals on all electrodes. The EOG rejection procedure rejected EEG signals occurring during rotations of the eyeball from 0.9 deg inward to 1.5 deg downward of visual angle–the stimulus spanned 5.36 deg×3.7 deg of visual angle on the screen. Artifact-free trials were sorted using EEProbe (ANT) software, narrow-band notch filtered at 49–51 Hz and re-referenced to average reference. For each electrode, EEG was measured every 3.9 ms, from −0.5 to 1 s around stimulus onset.

### Computation: Behavioral Classification Images

On each trial, randomly located Gaussian apertures make up a three-dimensional mask that reveals a sparse facial expression. Observers will correctly categorize the stimulus when the sampled SF information is diagnostic of the considered expression (e.g. revealing the wide-opened mouth in “happy.”) To identify the diagnostic SF features, we computed independently for each pixel the probability of being correct with this pixel by summing the aperture masks leading to correct categorizations and dividing the result by the sum of all aperture masks for that expression during the experiment (i.e. for correct and incorrect categorizations). This is analogous to performing a least-square multiple regression. We transformed these probabilities into Z-scores to locate the statistically significant pixels (p<.05, corrected, Pixel Test [46]). This procedure was carried out in each one of the five SF bands to represent the combination of SF bands and image features diagnostic for each expression (see Panel B of Figure S1 for an example of a behavioral classification image and its split of information across the five SF bands).

### Computation: Sensor-Based EEG Classification Images

To determine the facial features systematically correlated with modulations of the EEG signals, we applied Bubbles to EEG voltages to compute classification images, independently for each expression, sampled SF band, electrode, every 3.9 ms time point between −0.5 to 1 s around stimulus onset. Each classification image represented the subtraction of two sums: the sum of all bubble masks leading to amplitudes above (vs. below) the mean voltage, at this time point. We repeated the procedure for each one of the five spatial frequency bands and for each one of the seven expressions and each one of the 250 time points. For each electrode, this produced one classification image per SF band, time point and expression. Each classification image represents the significant (p<.05, corrected, [46]) facial information (if any) that is correlated with modulations of the EEG signal for that SF band, time point and expression (see [17],[23],[27],[28] for further details). Panel C of Figure S1 provides examples of EEG classification images on sensors OTR (in blue), OTL (in red) and POz (in green), over the time periods of the N170 and P300.

#### Information maximum

For each face pixel, we summed across the 5 SF bands the measure described in 5. Computation: Cycles per Face of Information in SF Bands and computed the location of the information maximum across the face.

#### Electrode selection

To reduce the dimensionality of the analysis, from the initial 58 electrodes, we focused on the subset of 19 equidistant electrodes that provide full coverage of the scalp (an adapted version of the 10/20 configuration, see Figure S2, Panel A). We chose to further analyze the electrodes OTR and OTL as these electrodes showed the typical N170 Event Related Potentials for the three observers (see Figure S2, Panel B). We chose the centro-parietal electrode (Pz/POz), as it showed sensitivity to the P300.

#### Region of interest analysis in figure 3

**Figure 3:**
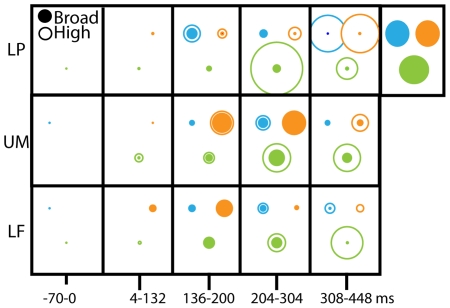
Trimming representations of diagnostic facial features: whole scalp. For each observer and expression, we computed over a subset of 19 electrodes covering the scalp (including OTR, OTL and Pz) the number of times an EEG classification image represented one of three regions of interest (the left eye, color-coded in cyan; the right eye, in orange and the mouth, in olive). When this information was diagnostic of the expression we computed its SF code. We classified each SF code as High SF when it was a combination of bands 1 to 3, binary codes [1]–[7], and as Broad SF if it was any other code. We then summed the relative numbers of High and Broad SF codes in five time windows [−70 to 0 ms; 4–132 ms; 136–200 ms; 204–304 ms and 308 to 448 ms] and represented the relative frequencies of High and Broad SF codes with the radii of open and filled circles. For each region of interest, features initially represented with High and Broad SF codes over the N170 time course become predominantly represented with HSF codes over the P300. The expansion of empty circles together with the contraction of filled circles illustrate that the trimming process is pervasive in the brain, not restricted to a few electrodes.

The region of interest analysis divided the face pixel space into three regions corresponding to the locations of the left eye, right eye, and mouth and counted when the information maximum of each EEG classification image fell inside those regions. We show the detailed results of this analysis in Table S1 and summarized the main outcomes in Figure 3.

### Computation: Comparison of EEG and Behavioral Classification Images

For each expression and electrode, we filtered the classification image at each time point, for each expression, and each observer, with the location map of information required for behavior and then constructed a cumulative sum of *new* information that overlapped with behavior (see Panel B of Figure 1). We examined the time course of this cumulative function, and found that all the information about the stimulus on OTR and OTL was added well before the peak of the N170. The derivative of this function had a clear maximum for each electrode, early in the course of N170, after which time little more information was added. The maximum of the derivative of the cumulative function is plotted as a triangle in panel A of Figure 1 and Figure S3 and S4.

### Computation: Spatial Frequency Coding

To examine the dynamics of spatial frequency usage in the brain, we examined the thresholded classification images for each for each time point, electrode, expression, and observer, and represented the specific combination of SF bands at each time point with a binary code (corresponding to decimal values comprised between 1 and 31, we show this code on the axis of all the binary coding figures). To illustrate, in Figure S1 C we show examples of broad SF content, representative of processing in the N170 (in blue and red), coded as 11111, indicating contribution of all bands. We also show an example of a ‘trimmed’ HSF classification image (in green), coded as 00001, taken from the P300. We thus created a binary code axis and represented transformations of SF composition over time (see Panel C of Figure 1 for OTR, OTL and POz). For OTR, OTL, and POz, for each observer and expression, we summarized the trajectory of SF codes over the time period with a cubic fit (R^2^ ranging from 0.2 to 0.66). To capture the overall transformation of SF composition across the scalp, we took all the points that fell into diagnostic regions of interest in the ROI analysis across 19 electrodes, and classified the SF composition as either high (codes 1–7) or broad (8–31), and report the outcome in Figure 3.

### Computation: Cycles per Face of Information in SF Bands

To understand how a combination of spatial frequency bands represents a feature, we derived a relative distribution of information (i.e. cycles per face). Specifically, at each time point, and on each electrode, we computed the total number of cycles per face represented in the statistically significant regions of the EEG classification image and summed cycles per face across the five SF bands. We then normalized the cycles per face measurements, to obtain for each time point, SF band, electrode, and expression a measure of information varying between 0 and 1 for each observer, and averaged across expressions. We then plotted the distribution of information per band over time, using line thickness to represent the different bands. Panel D of Figure 2 illustrates this plot. In addition Figure S5 shows the time course for expressions Fear and Happy for each observer.

## Supporting Information

Figure S1 Bubbles Methods Applied to Behavior and EEG Signals. Panel A: Bubbles Sampling. We decomposed the original image into five non-overlapping SF bands of one octave each (120−60, 60−30, 30−15, 15−7.5 and 7.5−3.8 cycles/face). To each SF band, we then applied a mask punctured with Gaussian apertures. These were positioned in random locations, trial by trial, approximating a uniform sampling of all face regions across many trials. The size of the apertures was adjusted for each SF band, so as to reveal 6 cycles per face. Calibration of the sampling density (i.e. the number of bubbles) was performed online on a trial-by-trial basis, to maintain observer's performance at 75% correct categorization, independently for each expression. The stimulus presented on each trial comprised the randomly sampled information from each SF band summed together, as shown. Panel B: Behavior Classification Image. In each sampled SF band the observer exploits features (e.g. the eyes and some mouth) to correctly classify the stimulus. We add this information across the five SF bands to derive the behavior classification image. This behavioral information can be used to examine when the EEG signals becomes sensitive to relevant behavioral information. Panel C: EEG Classification Images. Every 3.9 ms, we compute an EEG classification image on each of the 58 electrodes (illustrated midway through the N170 for OTR and OTL, in blue and red boxes and during the P300 for POz, in the green box). We assign a binary code to each EEG classification image (here, color-coded per electrode), representing the specific combination of SF bands in that particular image. For example, ‘11111’ indicates that all SF bands represent the left eye on blue OTR whereas ‘00001’ indicates that only the highest SF band represents the left eye and some of the mouth on green POz. We report in a plot color-coded per electrode, the sum of facial information (encoded as a normalized number of cycles per face) represented in each SF band–plotted here as different line thicknesses over the time course.
(8.45 MB TIF)

Figure S2 Average EEG and Spatial Frequency Composition of Classification Images as Scalp Topographies for Observers LP, UM and LF. Panel A: Electrode Locations. We show the layout of all 58 electrodes over the scalp, (black pixel squares), with the location of the electrodes selected for further analysis named in white. These 19 equally spaced electrodes cover the scalp in an adapted 10/20 configuration, including OTR, OTL and POz. Panel B: Average EEG. EEG signals on OTR, OTL and POz averaged over all 21 000 trials for each observer. Panel C: Scalp Topographies. For each observer, we constructed scalp topographies for the entire time course, representing the spatial frequency composition most frequently observed across all expressions at each time point on each electrode. In Panel C, we use a color code, where pale yellow indicates high spatial frequencies only, and red indicates full signal. We show the key transition from Broad to High SF, during the N170, between 133 and 234 ms following stimulus onset. Although the transition is centered on OTR (P8, blue circle), and OTL (PO7, red circle), the transition to HSF, shown as a change in color from red to yellow) is apparent on all active electrodes, as is the shift to the central-occipital region (Pz, POz, marked in green), as the N170 ends.
(7.96 MB TIF)

Figure S3 Trimming Representations of Diagnostic Facial Features (Observer UM, ‘Happy’ and ‘Fear’). Panel A: Average EEG. Colored curves represent the average EEG over PO7 (OTL, red), P8 (OTR, blue) and POz (green), while dashed curves represent the variance of the EEG. The upward triangle represents the point of maximum of EEG variance. On OTR and OTL this point is close to the upside-down triangle, (the maximum of the derivative of information accumulation shown on Panel B) where most novel, behaviorally relevant information is added to the classification images. Panel B: Behavior and Brain Feature Content. The gray-level background faces represent the facial features required for categorization behavior. Color-coded dots represent OTL and OTR sensors in red and blue; POz in green. Their Y coordinates, in correspondence with the behavioral features, reveal the location of the maximum of information of the EEG classification image at each time point. The color-coded curves plot the time course of accumulation of new information relevant for behaviour on each electrode. Most new information arrives during the early N170, indicating that most processing after the N170 is re-organization of inputs already taken up. Panel C: Spatial Frequency Composition. For each dot of panel B, a corresponding colored dot represents the binary-coded combination of SF bands of this particular feature at this time point. The colored dots trace a systematic upward trajectory over time in the binary codes, summarized, in yellow, with a cubic fit to those points containing diagnostic information. The trajectories illustrate that the same diagnostic features evolve from involving most SF bands (between 136 and 200 ms) to involving only the highest SF bands (between 200 and 448 ms).
(8.85 MB TIF)

Figure S4 Trimming Representations of Diagnostic Facial Features (Observer LF ‘Happy’ and ‘Fear’). Panels A–C as in Figure S3
(9.29 MB TIF)

Figure S5 Facial Information Content per Spatial Frequency Band. For each observer, we show example distributions of the respective contribution of each spatial frequency band (thickest line represents lowest Spatial Frequency band) over time. Line height illustrates the quantity of information (expressed in cycles per face) present in the classification image at this time point. This panel illustrates that all Spatial Frequency bands initially contribute information on the OTL and OTR (in red and blue) classification images. In contrast, only higher SFs contribute information to the late classification images on POz (in green). The distribution for each expression is similar to the average, shown in the main text.
(3.56 MB TIF)

Figure S6 Relative Percentage Distribution of SF codes. Panel A: Classification of the SF codes. The codes on the binary scale are classified in three ways. In yellow and labeled HSF, the first seven codes represent all combinations of the three HSF bands, indicating sensitivity to information in the upper three octaves (above 15 cycles per face). In light orange and labeled LSF, the next codes represent sensitivity to the two low spatial frequency bands, information below 15 cycles per face. The remaining codes labeled Broad, coded in dark orange indicate sensitivity to any other combination of SFs. Panel B: Trimming is a Shift from both Broad and Low to High Spatial Frequencies. For each observer, we classified the codes per region of interest from Table 1 as either HSF (represented in yellow); LSF (represented in orange) and Broad (shown as red). The colored vertical bars in Panel B represent the percentage of each class of code over the N170 time course. The flanked colored dots represent the proportions of these codes in the other time bins, relative to the N170 (i.e. represented as a percentage increase). For example, Observer LP shows a relative increase in HSF codes after the N170 (up to 157%), illustrating an increase in sensitivity to this information after the N170 peak. For each observer Panel B illustrates a substantial decrease of LSF and an almost complete loss of broad SF representation after the N170 peak. In contrast, HSF sensitivity is at least sustained.
(8.94 MB TIF)

Table S1 Region of Interest Analysis for 19 Electrodes. For each observer, the table reports the absolute (Nr. SF codes, bottom row) and relative (%ROI, top row) frequencies of SF codes covering the three region of interest considered. Each SF code corresponds to the information maximum of a classification image, and we pool the count over 19 electrodes. The table illustrates that following stimulus onset, most of the SF codes correspond to the SF encoding of diagnostic features representing left eye, right eye and mouth information.
(0.05 MB DOC)
