# Supplementary figures and images for: Dynamics of Trimming the Content of Face Representations for Categorization in the Brain
Source: PLoS Comput Biol. 2009 Nov 13;5(11):e1000561. doi: 10.1371/journal.pcbi.1000561 (PMC2768819; doi:10.1371/journal.pcbi.1000561)

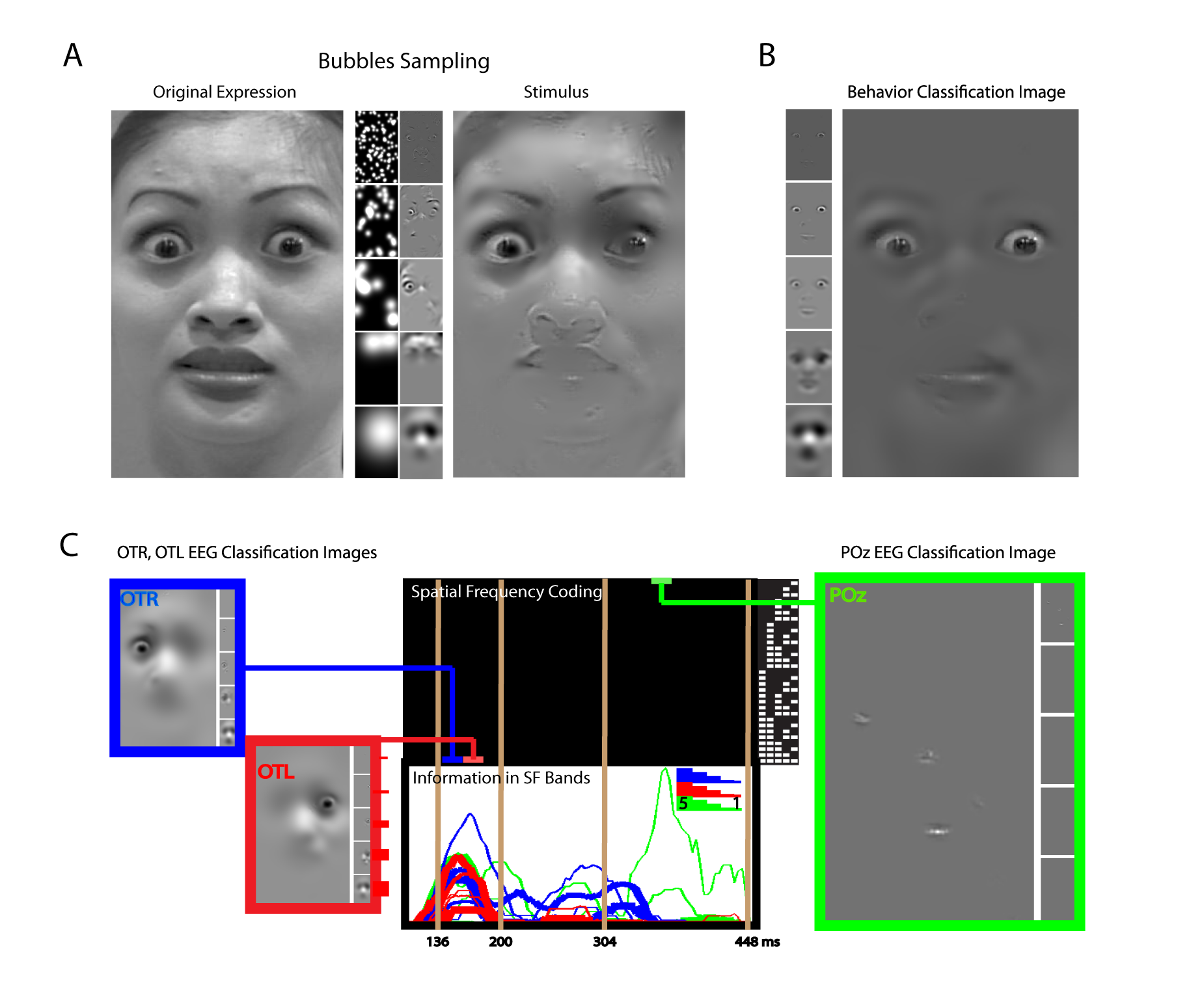

Supplement: Figure S1 — Bubbles Methods Applied to Behavior and EEG Signals. Panel A: Bubbles Sampling. We decomposed the original image into five non-overlapping SF bands of one octave each (120−60, 60−30, 30−15, 15−7.5 and 7.5−3.8 cycles/face). To each SF band, we then applied a mask punctured with Gaussian apertures. These were positioned in random locations, trial by trial, approximating a uniform sampling of all face regions across many trials. The size of the apertures was adjusted for each SF band, so as to reveal 6 cycles per face. Calibration of the sampling density (i.e. the number of bubbles) was performed online on a trial-by-trial basis, to maintain observer's performance at 75% correct categorization, independently for each expression. The stimulus presented on each trial comprised the randomly sampled information from each SF band summed together, as shown. Panel B: Behavior Classification Image. In each sampled SF band the observer exploits features (e.g. the eyes and some mouth) to correctly classify the stimulus. We add this information across the five SF bands to derive the behavior classification image. This behavioral information can be used to examine when the EEG signals becomes sensitive to relevant behavioral information. Panel C: EEG Classification Images. Every 3.9 ms, we compute an EEG classification image on each of the 58 electrodes (illustrated midway through the N170 for OTR and OTL, in blue and red boxes and during the P300 for POz, in the green box). We assign a binary code to each EEG classification image (here, color-coded per electrode), representing the specific combination of SF bands in that particular image. For example, ‘11111’ indicates that all SF bands represent the left eye on blue OTR whereas ‘00001’ indicates that only the highest SF band represents the left eye and some of the mouth on green POz. We report in a plot color-coded per electrode, the sum of facial information (encoded as a normalized number of cycles per face) represented in eac [file pcbi.1000561.s001.tif]

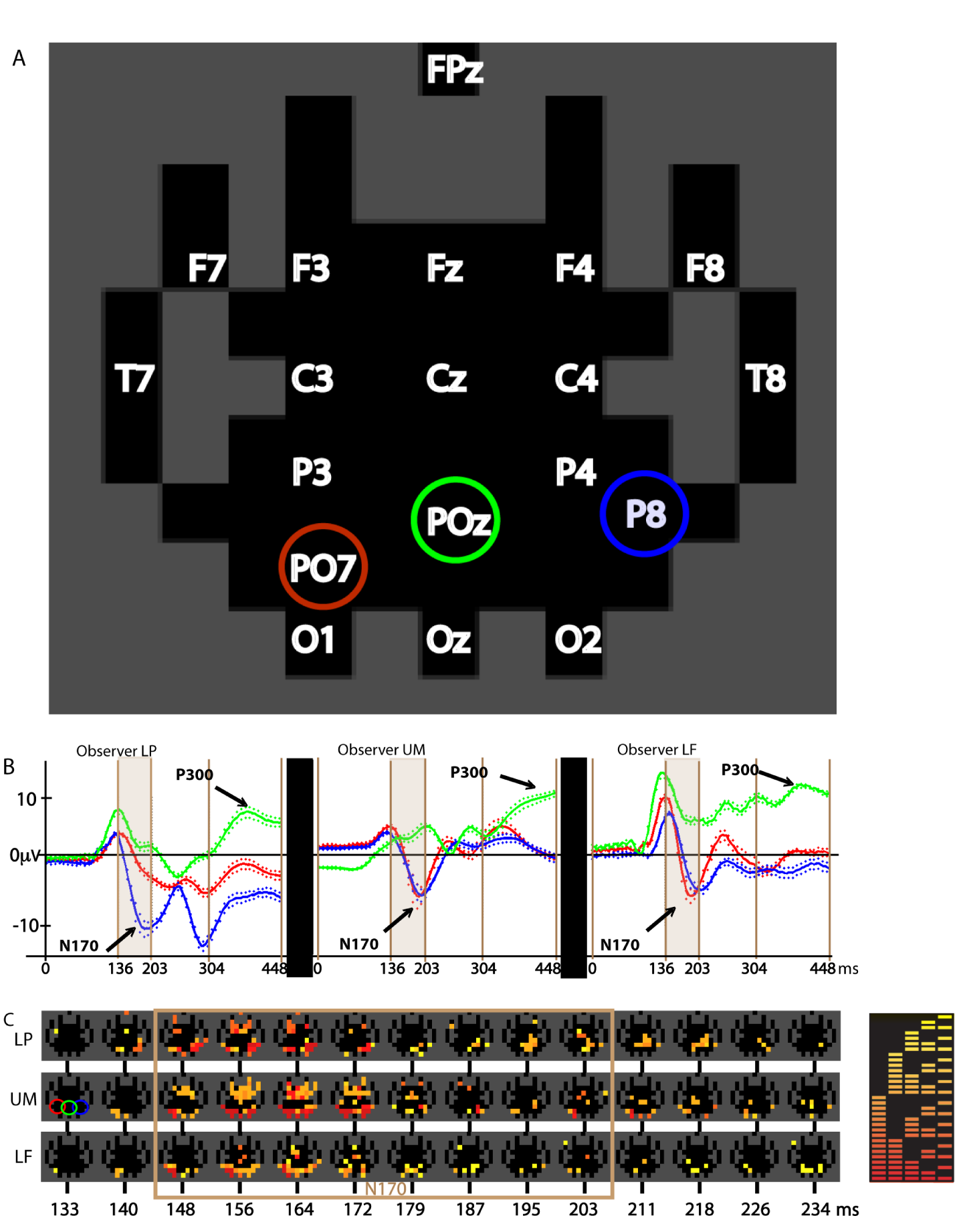

Supplement: Figure S2 — Average EEG and Spatial Frequency Composition of Classification Images as Scalp Topographies for Observers LP, UM and LF. Panel A: Electrode Locations. We show the layout of all 58 electrodes over the scalp, (black pixel squares), with the location of the electrodes selected for further analysis named in white. These 19 equally spaced electrodes cover the scalp in an adapted 10/20 configuration, including OTR, OTL and POz. Panel B: Average EEG. EEG signals on OTR, OTL and POz averaged over all 21 000 trials for each observer. Panel C: Scalp Topographies. For each observer, we constructed scalp topographies for the entire time course, representing the spatial frequency composition most frequently observed across all expressions at each time point on each electrode. In Panel C, we use a color code, where pale yellow indicates high spatial frequencies only, and red indicates full signal. We show the key transition from Broad to High SF, during the N170, between 133 and 234 ms following stimulus onset. Although the transition is centered on OTR (P8, blue circle), and OTL (PO7, red circle), the transition to HSF, shown as a change in color from red to yellow) is apparent on all active electrodes, as is the shift to the central-occipital region (Pz, POz, marked in green), as the N170 ends. (7.96 MB TIF) [file pcbi.1000561.s002.tif]

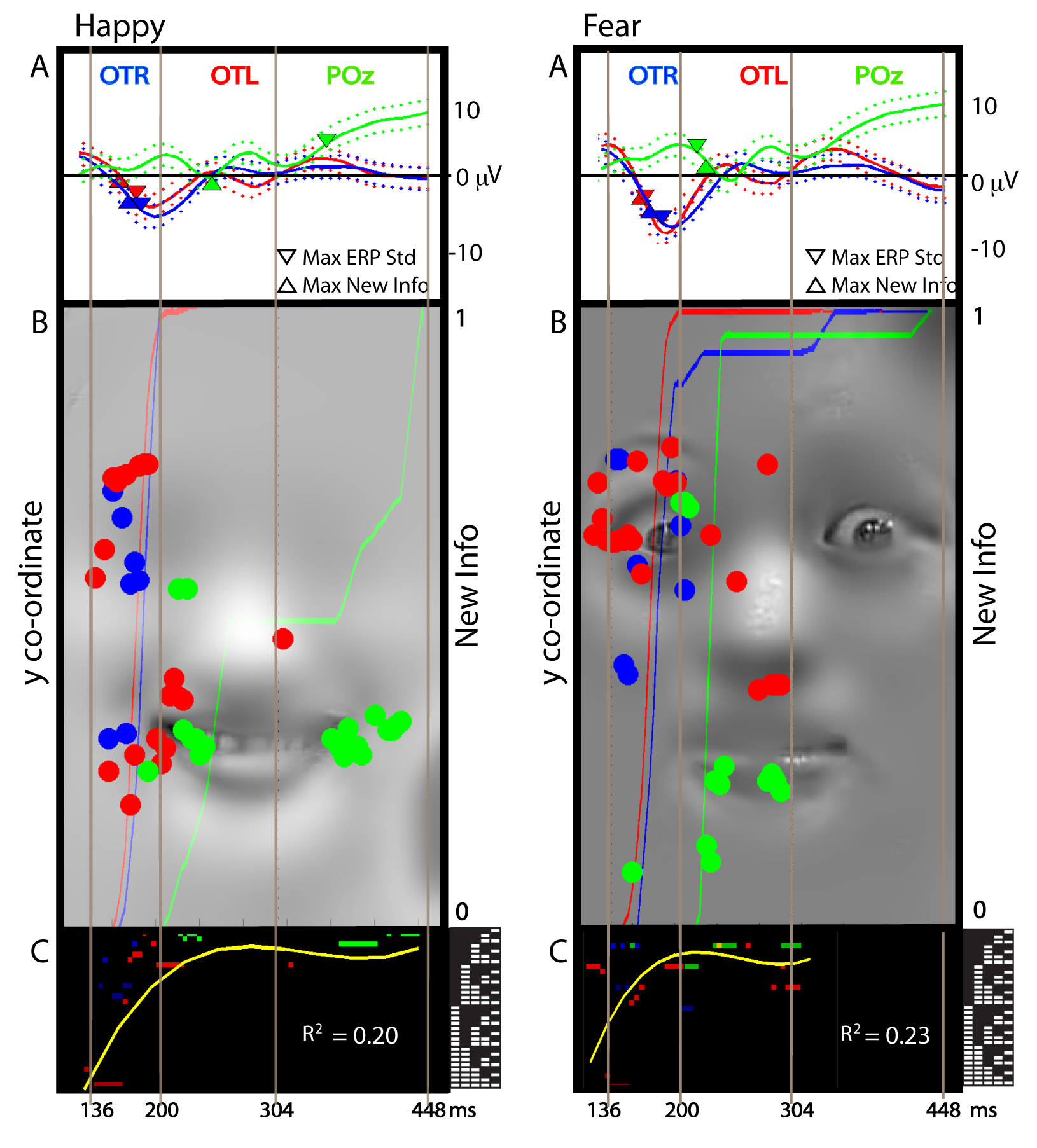

Supplement: Figure S3 — Trimming Representations of Diagnostic Facial Features (Observer UM, ‘Happy’ and ‘Fear’). Panel A: Average EEG. Colored curves represent the average EEG over PO7 (OTL, red), P8 (OTR, blue) and POz (green), while dashed curves represent the variance of the EEG. The upward triangle represents the point of maximum of EEG variance. On OTR and OTL this point is close to the upside-down triangle, (the maximum of the derivative of information accumulation shown on Panel B) where most novel, behaviorally relevant information is added to the classification images. Panel B: Behavior and Brain Feature Content. The gray-level background faces represent the facial features required for categorization behavior. Color-coded dots represent OTL and OTR sensors in red and blue; POz in green. Their Y coordinates, in correspondence with the behavioral features, reveal the location of the maximum of information of the EEG classification image at each time point. The color-coded curves plot the time course of accumulation of new information relevant for behaviour on each electrode. Most new information arrives during the early N170, indicating that most processing after the N170 is re-organization of inputs already taken up. Panel C: Spatial Frequency Composition. For each dot of panel B, a corresponding colored dot represents the binary-coded combination of SF bands of this particular feature at this time point. The colored dots trace a systematic upward trajectory over time in the binary codes, summarized, in yellow, with a cubic fit to those points containing diagnostic information. The trajectories illustrate that the same diagnostic features evolve from involving most SF bands (between 136 and 200 ms) to involving only the highest SF bands (between 200 and 448 ms). (8.85 MB TIF) [file pcbi.1000561.s003.tif]

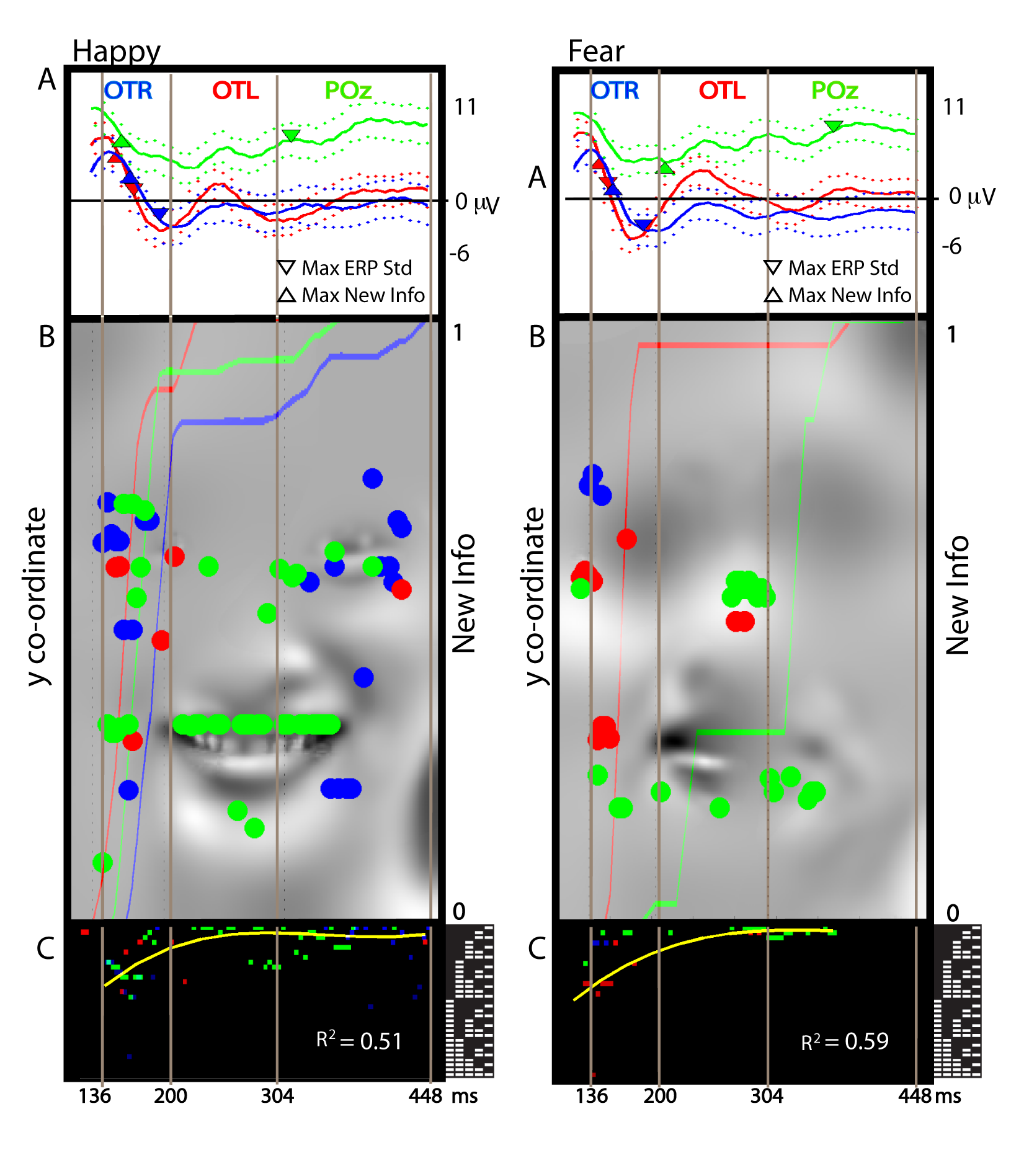

Supplement: Figure S4 — Trimming Representations of Diagnostic Facial Features (Observer LF ‘Happy’ and ‘Fear’). Panels A–C as in Figure S3 (9.29 MB TIF) [file pcbi.1000561.s004.tif]

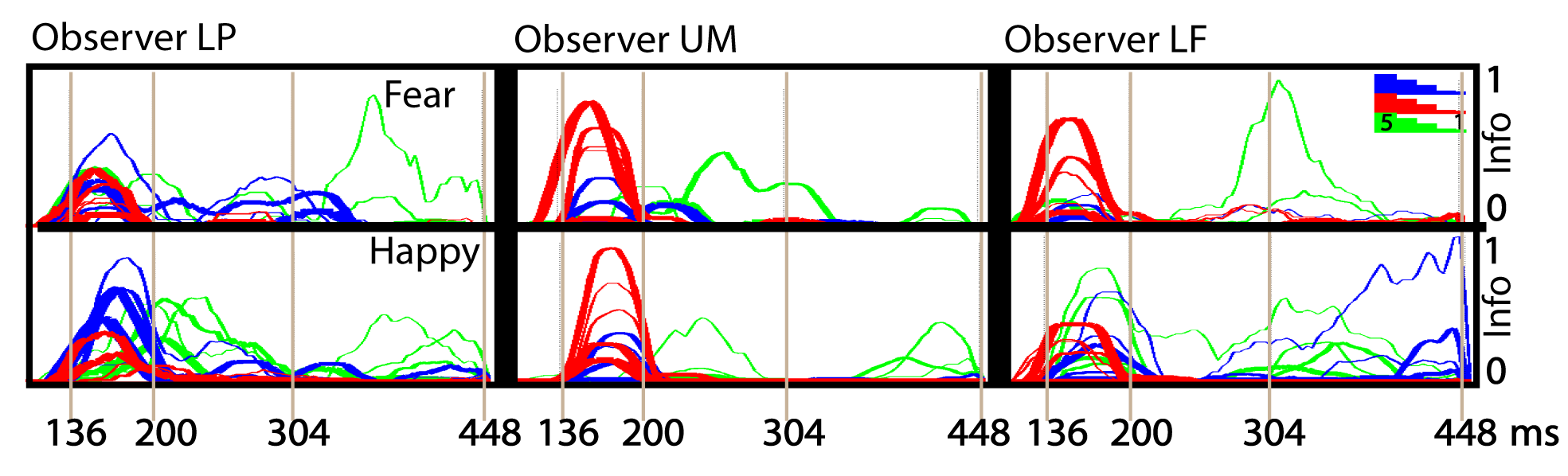

Supplement: Figure S5 — Facial Information Content per Spatial Frequency Band. For each observer, we show example distributions of the respective contribution of each spatial frequency band (thickest line represents lowest Spatial Frequency band) over time. Line height illustrates the quantity of information (expressed in cycles per face) present in the classification image at this time point. This panel illustrates that all Spatial Frequency bands initially contribute information on the OTL and OTR (in red and blue) classification images. In contrast, only higher SFs contribute information to the late classification images on POz (in green). The distribution for each expression is similar to the average, shown in the main text. (3.56 MB TIF) [file pcbi.1000561.s005.tif]

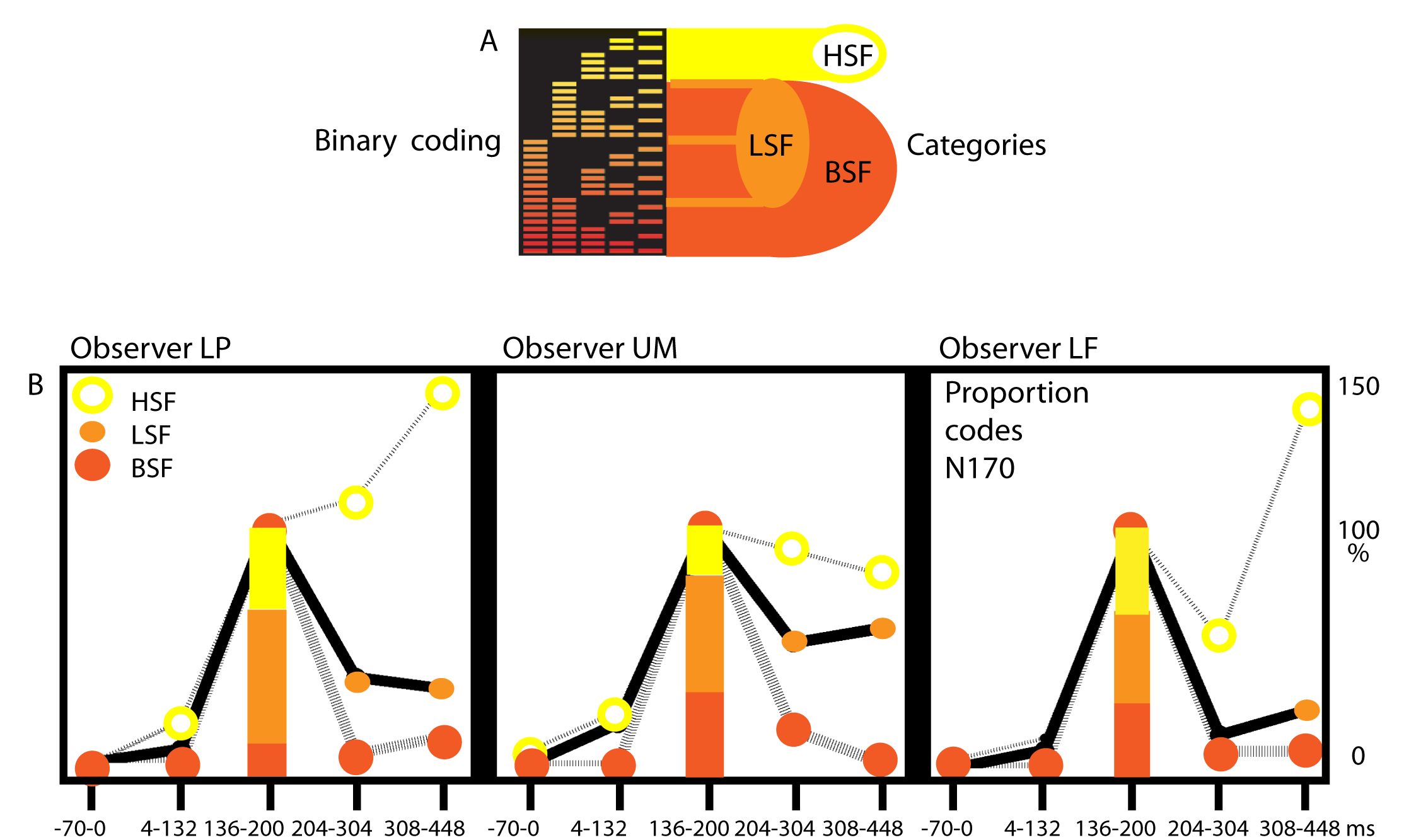

Supplement: Figure S6 — Relative Percentage Distribution of SF codes. Panel A: Classification of the SF codes. The codes on the binary scale are classified in three ways. In yellow and labeled HSF, the first seven codes represent all combinations of the three HSF bands, indicating sensitivity to information in the upper three octaves (above 15 cycles per face). In light orange and labeled LSF, the next codes represent sensitivity to the two low spatial frequency bands, information below 15 cycles per face. The remaining codes labeled Broad, coded in dark orange indicate sensitivity to any other combination of SFs. Panel B: Trimming is a Shift from both Broad and Low to High Spatial Frequencies. For each observer, we classified the codes per region of interest from Table 1 as either HSF (represented in yellow); LSF (represented in orange) and Broad (shown as red). The colored vertical bars in Panel B represent the percentage of each class of code over the N170 time course. The flanked colored dots represent the proportions of these codes in the other time bins, relative to the N170 (i.e. represented as a percentage increase). For example, Observer LP shows a relative increase in HSF codes after the N170 (up to 157%), illustrating an increase in sensitivity to this information after the N170 peak. For each observer Panel B illustrates a substantial decrease of LSF and an almost complete loss of broad SF representation after the N170 peak. In contrast, HSF sensitivity is at least sustained. (8.94 MB TIF) [file pcbi.1000561.s006.tif]
