# Supplementary material for: Dynamics of Trimming the Content of Face Representations for Categorization in the Brain
Source: PLoS Comput Biol. 2009 Nov 13;5(11):e1000561. doi: 10.1371/journal.pcbi.1000561 (PMC2768819; doi:10.1371/journal.pcbi.1000561)
Supplement: Table S1 — Region of Interest Analysis for 19 Electrodes. For each observer, the table reports the absolute (Nr. SF codes, bottom row) and relative (%ROI, top row) frequencies of SF codes covering the three region of interest considered. Each SF code corresponds to the information maximum of a classification image, and we pool the count over 19 electrodes. The table illustrates that following stimulus onset, most of the SF codes correspond to the SF encoding of diagnostic features representing left eye, right eye and mouth information. (0.05 MB DOC) [file pcbi.1000561.s007.doc]

| Observers | Time bin (ms) | -70 to 0 | 0 to 132 | 136 to 200 | 204 to300 | 304 to 448 |
| --- | --- | --- | --- | --- | --- | --- |
| LP | % ROI | 33% | 50% | 67% | 85% | 71% |
|  | Nr. SF codes | 1/3 | 9/18 | 134/199 | 204/245 | 176/249 |
| LF | % ROI | 44% | 72% | 71% | 70% | 57% |
|  | Nr. SF codes | 4/9 | 13/18 | 170/241 | 130/183 | 160/283 |
| UM | % ROI | 50% | 76% | 74% | 76% | 73% |
|  | Nr. SF codes | 2/4 | 25/33 | 250/332 | 298/392 | 175/240 |

**Table S1**: **Region of Interest Analysis for 19 Electrodes.** For each observer, the table reports the absolute (Nr. SF codes, bottom row) and relative (%ROI, top row) frequencies of SF codes covering the three region of interest considered. Each SF code corresponds to the information maximum of a classification image, and we pool the count over 19 electrodes. The table illustrates that following stimulus onset, most of the SF codes correspond to the SF encoding of diagnostic features representing left eye, right eye and mouth information.
